# Deposit of microbial strains in public service collections as part of the publication process to underpin good practice in science

**DOI:** 10.1186/2193-1801-3-208

**Published:** 2014-04-28

**Authors:** Erko Stackebrandt, David Smith, Serge Casaregola, Giovanna Cristina Varese, Gerard Verkleij, Nelson Lima, Paul Bridge

**Affiliations:** MIRRI, c/o DSMZ, Braunschweig, Germany; CABI, Egham, Surrey, TW20 9TY UK; CIRM-Levures Micalis, Inra/AgroParisTech, 78850 Thiverval-Grignon, France; MUT - Department of Life Science and Systems Biology, University of Turin, 10125 Torino, Italy; CBS-KNAW Fungal Biodiversity Centre, Uppsalalaan 8, 3584 CT Utrecht, Netherlands; University of Minho, Braga, Portugal

**Keywords:** Biological resource centres, Deposition policy, Credibility of science, Deposition ‘key’ criteria

## Abstract

Despite recommendations to release microbial resources to the community post-publication, the reality is far from satisfying. A workshop discussed the need for a coordinated and effective deposition policy for ‘key’ microbial strains and proposes a set of criteria to facilitate their deposition into public service collections. The majority of authors either contacted directly or during submission of manuscripts to several international, mainly European bacteriology journals agreed to this set of ‘key strain’ criteria and to the voluntarily deposition of resources into public resource centres.

**Electronic supplementary material:**

The online version of this article (doi:10.1186/2193-1801-3-208) contains supplementary material, which is available to authorized users.

## Findings

As stated in the “Instructions to Authors” of almost all scientific journals, material, data and protocols should be made available in order to allow others to “replicate and build upon the authors’ published claims” (Anonymous 2014). While deposition of genomic sequences in public databases and of type strains of validly named prokaryotic species in public resource centers are two examples for a functioning implementation, deposition and release of material and data are, in practise, frequently left to the authors’ discretion. Some journals may have a stricter implementation policy than others but generally enforcement mechanisms do not exist in those frequent cases where authors deny release of requested material by either not responding or by stating, among other arguments, intellectual property priorities, loss of material, biosecurity issues, export and import regulations, or patent issues. An alternative option to direct requests to authors for strains is the deposition of material in public collections, following the example of Nature journals, which request deposition of mutants and cell lines in well recognized repositories (Anonymous 2014).

The motivation to organize a workshop on enhanced access on post-publication microbial resources was a recent survey on frequency of deposition of prokaryotic strains (Stackebrandt 2010). Workshop participants confirmed that, though access to published material may work smoothly among scientists in certain disciplines and tightly knit scientific communities, access is not widespread. Also, the deposition in collections of strains that are cited in the literature to facilitate their availability, to confirm findings and extend studies, is dismal (Stackebrandt 2010). Of 20,200 prokaryotic strains reported in 835 articles in eight European journals in 2008, only 190 strains (0.94%) were deposited in public collections. What are the reasons behind this and why are the huge investments in publicly funded research not protected for confirmation of results and future use? It is evident that the biological materials on which published data has been generated must be available to check when aberrant or erroneous results are discovered or when new technologies are available for further study and characterization.

The low level of deposition of prokaryotic strains into today’s public service collections (the microbial domain Biological Resource Centre - mBRC), and bad experiences of participants when attempting to access biological resources post publication, triggered discussion on a strategy to enhance and facilitate access to microbial resources. This was done whilst being aware that deposition of all microbial strains is not achievable under the present funding system of public repositories (Stackebrandt 2010). Against this background, we recommend a set of selection criteria that would allow all stakeholders to prioritize material for deposition.

Public service culture collections have been performing this function for over 120 years. There are currently about 2,391 million microbial strains available in the 660 collections listed in the World Data Centre for Microorganisms (http://www.wdcm.org). There are about 694,000 fungi representing the approximately 100,000 fungal species described (Rossmann 1995), and about 1,024,000 bacteria and archaea representing the 10,600 or so validly named species (http://www.bacterio.net/). The repositories provide the optimal environment for long-term maintenance and curators are experts in identification, regulatory and biosafety issues and international shipping of microbial resources. In academic or other research environments, maintenance cannot be assured and resources are prone to loss or death. Collections can react much more quickly and professionally than other facilities to the emergence of novel microbiological material. Collections are experienced in the deposition of type strains and more so of an enormous number of non-type strain deposits but there are still obvious and considerable gaps in the availability of representative strains of the species given the immense variation of expressed properties of strains. These gaps, especially those occurring in rare and underrepresented taxa, could in part be filled by enhanced deposition of strains as part of the publication process for the article.

The offer of major West European public service collections, combined in the EU projects MIRRI (http://www.mirri.org), EMbaRC (http://embarc.eu) and the GBRCN collection consortium (http://www.gbrcn.org) to accept resources during or shortly after publication is a spark to ignite the participation of other such collections worldwide. The rationale for doing so is not based on a concern that authors are incapable of short-term handling of research strains; it is based on the fact that microbial resource centres have decades of experience in handling, safeguarding and shipping a wide range of diverse material that is otherwise prone to involuntary loss by negligence or deliberate clearing of laboratory holdings. The public service collections/mBRCs comply with applicable regulatory requirements, provide material under material transfer agreements settling terms and conditions of supply and governing intellectual property.

In addition to the safeguarding of this biological material, access to these resources will be facilitated; all recognized mBRCs maintain easily accessible databases, in which strains and associated data including origin of the strains as well as depositor and cognate publications are searchable. Initiatives that allow searches among the resources and data of several mBRCs simultaneously to make them more consultable (CABRI, WDCM and in the future MIRRI-Information System) have been undertaken to widen the access to these resources making them more likely to be utilized in research and thus increasing the chances to develop scientific collaborations and industrial exploitation of the resources.

The workshop participants decided against a mandatory post-publication deposition of microbial strains but agreed on a set of criteria based on the phylogenetic, metabolic and genomic uniqueness of “key” strains worthy of deposit. These criteria apply for isolates from basic and applied research and in case of large isolation study both, authors and mBRC curators, should decide on the selection and number of strains to be deposited. A checklist for this process would be needed and would contain the contact addresses of a range of public service collections together with their taxonomic priorities to facilitate contact between authors and curators. Completion of this checklist would be mandatory prior to manuscript submission. The definition of ‘key’ strains should be seen as a first but not exclusive step to initiate the strain sharing strategy; environmental samples, including as-yet uncultured microorganisms, metagenome libraries and other material should also be considered medium-term. The following criteria were agreed:

For Prokaryotes:

- Phylogenetic uniqueness, based on a cutoff point of ≤98% of 16S rRNA gene sequence from its nearest phylogenetic neighbor. This sequence is the gold standard for phylogenetic affiliation of an isolate at the genus level;
- Metabolic uniqueness, based on the presence of a new pathway, modification of an existing pathway, metabolic differences compared to the type strain or novel products including any strains with demonstrated useful properties i.e. production of specific molecule, biopesticide, biofertilizer, degradation of specific compounds, etc. to facilitate biotech exploitation;
- Genomic uniqueness, such as significant differences (≥20%) in genome size, genome architecture or new regulatory mechanisms;
- Resources and parts thereof with fully sequenced genomes (microorganisms, phages, plasmids);
- A second strain of those species for which only the type strain has been described. 79% of new species described in 2009 are based on the type strain only (Stackebrandt 2010);
- Strains associated with significant or new plant and animal diseases in order to ensure reliable reference material is available for diagnostic services and activities;
- Strains from unexplored or extreme environments (e.g. naturally extreme environments, foodstuffs, polluted environments) to reduce the gaps in the holdings, to study adaptation etc.…;

For fungi:

- (Ex-) type strains of novel taxa – currently there is not a mandatory process for storing living cultures of the dead dried reference material for fungal types;
- Phylogenetic uniqueness, based on significant differences in the various phylogenetic markers defined for yeasts and fungi (e.g. ITS, D1/D2, SSU, LSU, EF1-alpha, tubulin, etc.);
- Metabolic uniqueness, based on the presence of a new pathway, modification of an existing pathway, metabolic differences compared to the type strain or novel products; including any strains with demonstrated useful properties i.e. production of specific molecule, biopesticide, biofertilizer, degradation of specific compounds, etc. to facilitate biotech exploitation;
- Any strain associated to a complete (or partial) nuclear genome sequence (as a reference and/or as part of future population studies). This includes the genomic uniqueness criteria of the prokaryote list;
- Strains from population studies (to further estimate biodiversity in various niche, environment, substrate etc.…);
- Several strains of those species for which only the type strain has been described (to allow delineation of species and to find strains with opposite mating types for genetic experimentation and strain improvement);
- Strains from unexplored or extreme environments (naturally extreme environments, foodstuffs, polluted environments etc.) to reduce the gaps in the holdings, to study adaptation etc.;
- Strains associated with significant or new plant and animal diseases in order to ensure reliable reference material is available for diagnostic services and activities.

A survey on author reaction to voluntary deposition of ‘key’ strains was sent in 2011 to 3.900 authors in 49 countries of whom 503 authors (12.9%) responded. 437 authors (87%) of them agreed that there was a need to improve access to microbial resources and 397 authors (79%) agreed that journal publication guidelines should request that strains with particular properties, such as those listed in the above Boxes, must be deposited in public culture collections to maintain them for further research. When asked if they had encountered problems in accessing strains 301 authors (59.8%) of the responders indicated that they had frequent problems when asking authors for strains. Around 78% of those indicated that they often or never received a response at all, others were requested to pay for the strain and some were denied access because of patent issues.

These responses suggest that the responders believe that a behavioural change is necessary and that journals should request that strains associated with publications be deposited. The reasons given for lack of response or failure to receive strains (Additional file 1) were specifically indicated by about 100 scientists but are subject to conjecture, being a mixture of guesses and author citations. In approximately 39% of cases it is feared that researchers simply want to protect their research from exploitation by others. This appears to be the very opposite to the philosophy behind publication and dissemination of results and conflicts with accepted scientific principles and morals. About 31% referred to the authors response that strains were lost or were unavailable for non-specified reasons; 25% referred to quarantine, customs and biosafety regulations as severe obstacles for releasing strains, a problem that would be better solved by international, experienced microbial resource centers than by individual scientists. Authors and collection curators should work closely together to resolve the hurdles connected to shipment of microbial strains. Additionally, to protect the investment made using public funds, the research funders must also consider whether they make similar deposit and availability requirements. Governmental funding policies (Additional file 2 provides links for four agencies) specifically deal with data sharing policy rather than with the physical outputs of research. Deposition is not or only rarely mentioned.

In a follow-up activity, a 12-month survey was linked to the submission of manuscripts to 10 international, mainly European microbiology journals. Asked whether they are interested in deposition of ‘key’ strains 542 of the 890 responding authors (61%) agreed, 188 authors (21%) were undecided while only 160 authors (18%) denied.

The workshop participants stressed that:

- authors should make every reasonable effort to make material available;
- journals and funding agencies should monitor their policies and have a mechanism for accepting complaints where access to material is denied;
- journals should introduce a mechanism for active agreement by authors to make material available when they submit an article;
- microbial resource collections should develop strategies and secure funding for the expected need to expand infrastructure and personnel (Smith et al. 2014).

The workshop did not address the financial consequences of enhanced deposition but, considering the urgency to act now, funding agencies need to re-evaluate their responsibilities by providing long-term and increasing support for public repositories to allow these tasks to be performed (Emerson and Wilson 2009; Stackebrandt 2010; Stackebrandt 2011). There are an increasing number of journals that have policy that recommends the deposit of strains as part of the publication process including Antonie van Leeuwenhoek, Archives of Microbiology, Applied Microbiology and Biotechnology, FEMS Yeast Research, Fungal Biology, Fungal Genetics and Biology, International Journal of Systematic and Evolutionary Microbiology, Journal of General and Applied Microbiology, Medical Mycology, Mycologia, and Mycoses. It is hoped that for the sake of good science such policies are adopted by all journals.

## Electronic supplementary material

Additional file 1: **Evaluation of the questionnaire on author reasons for not sharing strains or not receiving strains from peers.** (DOCX 14 KB)

Additional file 2: **Funding bodies policy of NIH (USA), MRC and BBSRC (UK) and DFG (Germany) on sharing of research data.** (DOCX 13 KB)
