# Supplementary material for: Deposit of microbial strains in public service collections as part of the publication process to underpin good practice in science
Source: Springerplus. 2014 Apr 28;3:208. doi: 10.1186/2193-1801-3-208 (PMC4018474; doi:10.1186/2193-1801-3-208)
Supplement: Supplementary file 1 — Additional file 1: Evaluation of the questionnaire on author reasons for not sharing strains or not receiving strains from peers. (DOCX 14 KB) [file 40064_2014_929_MOESM1_ESM.docx]

**Deposit of microbial strains in public service collections as part of the publication process to underpin good practice in science**

Supplement 1

Evaluation of the questionnaire on author reasons for not sharing strains or not receiving strains from peers.

|  | How often (in numbers and % of responses) did you encounter problems when asking authors for microbial strains described in their papers? | | |
| --- | --- | --- | --- |
|  | Never to rarely  0-20% | Often  20-60% | Very often to always  60-100% |
| Problems encountered | **181 (37.5%)** | **185 (38.4%)** | **116 (24.1%)** |
| Identified reasons | | | |
| No response | **247 (51.3%)** | **185 (38.5%)** | **49 (10.2%)** |
| Transfer against charge | **303 (63.0%)** | **110 (22.9)** | **68 (14.1%)** |
| Denied:  Patent issues | **355 (76.0%)** | **74 (15.9%)** | **38 (8.1%)** |
| No access: other reasons | **382 (80.6%)** | **56 (11.8%)** | **36 (7.6%)** |
