# Supplementary material for: Deposit of microbial strains in public service collections as part of the publication process to underpin good practice in science
Source: Springerplus. 2014 Apr 28;3:208. doi: 10.1186/2193-1801-3-208 (PMC4018474; doi:10.1186/2193-1801-3-208)
Supplement: Supplementary file 2 — Additional file 2: Funding bodies policy of NIH (USA), MRC and BBSRC (UK) and DFG (Germany) on sharing of research data. (DOCX 13 KB) [file 40064_2014_929_MOESM2_ESM.docx]

**Deposit of microbial strains in public service collections as part of the publication process to underpin good practice in science**

Supplement 2

**Funding bodies policy of NIH (USA), MRC and BBSRC (UK) and DFG (Germany) on sharing of research data:**

http://www.mrc.ac.uk/Ourresearch/Ethicsresearchguidance/Datasharinginitiative/index.htm

http://grants.nih.gov/grants/guide/notice-files/NOT-OD-03-032.html

http://www.bbsrc.ac.uk/publications/policy/data_sharing_policy.html

http://www.dfg.de/download/pdf/foerderung/grundlagen_dfg_foerderung/informationen_fachwissenschaften/lebenswissenschaften/checkliste_gwa_studien.pdf
